# Discordance in CD4+T-Cell Levels and Viral Loads with Co-Occurrence of Elevated Peripheral TNF-α and IL-4 in Newly Diagnosed HIV-TB Co-Infected Cases

**DOI:** 10.1371/journal.pone.0070250

**Published:** 2013-08-01

**Authors:** Ronald Benjamin, Atoshi Banerjee, Sharada Ramaseri Sunder, Sumanlatha Gaddam, Vijaya Lakshmi Valluri, Sharmistha Banerjee

**Affiliations:** 1 Department of Biochemistry, School of Life Sciences, University of Hyderabad, Hyderabad, Andhra Pradesh, India; 2 Immunology, Molecular Biology & Biochemistry Division, LEPRA-India, Blue Peter Public Health & Research Centre, Cherlapally, Hyderabad, Andhra Pradesh, India; 3 Department of Immunology, Bhagawan Mahavir Medical Research Centre, Hyderabad, Andhra Pradesh, India; Seattle Biomedical Research Institute, United States of America

## Abstract

**Background:**

Cytokines are the hallmark of immune response to different pathogens and often dictate the disease outcome. HIV infection and tuberculosis (TB) are more destructive when confronted together than either alone. Clinical data related to the immune status of HIV-TB patients before the initiation of any drug therapy is not well documented. This study aimed to collect the baseline information pertaining to the immune status of HIV-TB co-infected patients and correlate the same with CD4+T cell levels and viral loads at the time of diagnosis prior to any drug therapy.

**Methodology/Principal Findings:**

We analyzed the cytokines, CD4+T cell levels and viral loads to determine the immune environment in HIV-TB co-infection. The study involved four categories namely, Healthy controls (n = 57), TB infected (n = 57), HIV infected (n = 59) and HIV-TB co-infected (n = 57) patients. The multi-partite comparison and correlation between cytokines, CD4+T-cell levels and viral loads prior to drug therapy, showed an altered TH1 and TH2 response, as indicated by the cytokine profiles and skewed IFN-γ/IL-10 ratio. Inadequate CD4+T cell counts in HIV-TB patients did not correlate with high viral loads and *vice-versa*. When compared to HIV category, 34% of HIV-TB patients had concurrent high plasma levels of IL-4 and TNF-α at the time of diagnosis. TB relapse was observed in 5 of these HIV-TB co-infected patients who also displayed high IFN-γ/IL-10 ratio.

**Conclusion/Significance:**

With these studies, we infer (i) CD4+T-cell levels as baseline criteria to report the disease progression in terms of viral load in HIV-TB co-infected patients can be misleading and (ii) co-occurrence of high TNF-α and IL-4 levels along with a high ratio of IFN-γ/IL-10, prior to drug therapy, may increase the susceptibility of HIV-TB co-infected patients to hyper-inflammation and TB relapse.

## Introduction

Human Immunodeficiency Virus (HIV) infection is a pandemic, with more than 34 million people infected worldwide (UNAIDS-2011). HIV weakens the human immune system, increasing the probability of other opportunistic pathogens to cause infections. One of the most prevalent co-infection in HIV patients is by *Mycobacterium tuberculosis* (*M.tb*), the TB causing bacteria. *M.tb* infection adds to the morbidity and mortality rates of HIV patients as there is an increased risk of disease progression when an individual is concurrently infected with *M.tb* and HIV [1], [2]. Yet another emerging issue is Immune Reconstitution Inflammatory Syndrome (IRIS). IRIS is a condition seen in certain cases of HIV infection where the compromised immune system begins to recover substantially after introduction of highly active anti-retroviral therapy (HAART), but in turn exhibits an overwhelming inflammatory response to a previous opportunistic infection that paradoxically makes the symptoms of infection worse [3]. Pro-inflammatory and regulatory cytokines play an important role in the immunopathology of HIV and TB infections. Although a wide range of cytokines may contribute to the protection, TH1 or pro-inflammatory response dominated by TNF-α, IFN-γ and IL-12 are the principle mediators of protective immunity against TB [4] but are permissive factors for HIV [5]–[6]. The TH2 response characterized by the secretions of IL-4 and IL-10 mediate resistance to HIV [5], [6], but is known to help activation of latent TB [7]. It is this delicate balance of TH1 and TH2 response which becomes the deciding factor for the progression of HIV and TB infections.

Destruction of CD4+T cells by HIV is a marker for the disease progression, which eventually makes the patients vulnerable to opportunistic infections leading to acquired immunodeficiency syndrome (AIDS) [8]. Yet another indicator of the disease progression is the HIV load, measured in terms of the number of copies of viral RNA in the peripheral blood. High plasma viral RNA copy numbers are closely linked with low CD4+T cell counts and are used as an indicator of the disease progression to AIDS [8]. CD4+T cell counts and viral loads are often relied on as the prognostic factors for the initiation of highly active anti-retroviral therapy (HAART) in HIV-TB co-infected patients undergoing TB therapy [8]. Concurrent drug therapies against HIV and TB in HIV-TB co-infected patients lead to complications due to drug cytotoxicity and high probability of hyper-inflammatory syndromes. Identifying the early symptoms in patients who are at high risk of hyper-inflammation can be a difficult task. However, deeper analyses of the immune status through cytokine levels along with the CD4+T cell counts and viral loads can help decipher the immune environment in a patient at the time of diagnosis.

Several studies in different populations have been performed to understand the cytokine response during HIV-TB co-infection. One of the studies revealed that the PBMCs of HIV-TB patients secrete less of IFN-γ and IL-12 when compared to TB patients after stimulation with mycobacterial antigens. However, these *in vitro* observations did not correlate with *in vivo* observations where higher levels of IFN-γ, IL-12 and IL-18 were detected in the plasma of HIV-TB patients [9], suggesting that the *in vitro* stimulation assays cannot be taken as a reflection of the response to HIV and TB infections under systemic conditions. Several studies have provided clues for the population variations in the cytokine responses [10]. Most often, HIV-TB co-infected patients included in these studies were already on anti-retroviral therapy (ART) at the time of detection of mycobacterial co-infection. Clinical studies related to the immune status of HIV-TB patients before the initiation of any drug therapy is limited. This study was aimed to collect baseline information pertaining to the immune status of HIV-TB co-infected patients and correlate the same with CD4+T cell counts and viral loads before the initiation of either anti-TB or anti-HIV treatments, assuming that the initial state of the immune environment may play a decisive role in the outcome of the drug therapies.

We studied the pro- and anti-inflammatory environment in a well characterized group of newly diagnosed HIV-TB co-infected patients before the onset of any drug regimen, in order to use these parameters as predictive markers for the outcome of the treatment, primarily TB treatment alongside HAART in HIV-TB co-infected patients. In our study, multi-partite comparisons and correlations were made between the cytokines, CD4+T cell levels and viral loads. The patients were monitored for the outcome of HIV and TB therapies till the completion of TB drug regimen and the cases of severe health deterioration and relapses were noted. We observed that in HIV-TB category, low CD4+T cell levels did not represent the disease progression in terms of increase in the viral load. HIV-TB co-infected patients neither had a well-defined pro-inflammatory TH1 (IL-12 p70, IFN-γ and TNF-α) nor a regulatory TH2 (IL-4 and IL-10) immune environment at the time of diagnosis, which was further evident from the intermediate IFN-γ/IL-10 ratio in these patients. 34% of HIV-TB patients at the time of diagnosis had high levels of both TNF-α and IL-4 in addition to the high ratio of IFN-γ/IL-10. Five of these HIV-TB co-infected patients had TB relapse after successful completion of TB treatment.

With this study, two inferences could be made; first, high CD4+T cell counts in HIV-TB patients did not indicate low plasma viral loads and hence may not be appropriate for HIV disease monitoring in HIV-TB patients. Second, high plasma levels of TNF-α and IFN-γ/IL-10 ratio at the onset of the detection of co-infection, indicated a pro-inflammatory environment in HIV-TB patients, thus increasing their probability of mounting a hyper-inflammation like syndrome after ART.

## Results and Discussions

### Discordance between CD4+T Cell Levels and Viral Loads in Newly Diagnosed HIV-TB Patients

**Figure 1:**
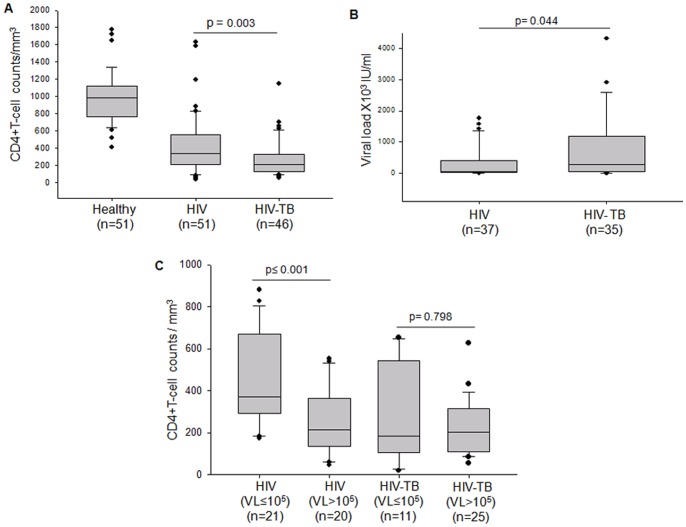
Correlation of CD4+T cells and viral load as indicators of HIV progression. Box plots representing (A) the distribution of CD4+T cell/mm^3^ of blood in Healthy, HIV and HIV-TB. (B) The distribution of viral RNA copies measured in terms of IU/ml of sample for the HIV and HIV-TB patients. (C) Analyses of the CD4+T cell count of HIV and HIV-TB samples with low viral load (VL≤10^5^ IU/ml) and high viral load (VL>10^5 ^IU/ml). The threshold for significance was set at p≤0.05. Bars above the plots represent the statistical significance (p value) between the groups.

The levels of CD4+T cells and viral loads were measured in HIV-TB co-infected patients and compared with that of HIV category. Similar to other studies, we observed that the CD4+T cell depletion was higher in HIV patients with TB infection as compared to the patients with HIV infection alone (p = 0.003) (Fig. 1A). The median value of CD4+T cell counts of the patients under HIV category was 336/mm^3^, whereas that for HIV-TB co-infection category was only 208/mm^3^ (Fig. 1A). T-lymphopenia was not observed in TB patients under our study, unlike reported in some studies [11].

Viral loads of patients under HIV and HIV-TB categories varied over a wide range; hence comparing the means for the two categories was not informative. The medians of the two groups were significantly different. In case of HIV, the median was 51.68×10^3^ IU/ml (IQR: 17.5×10^3^–407.1×10^3^ IU/ml) whereas that for HIV-TB category, the median was 269.1×10^3 ^IU/ml (IQR: 58.82×10^3^–1173.4×10^3^) (Fig. 1B). This clearly indicated that the HIV viral load was higher when there is a TB co-infection. There was more than twofold increase in the viral titers in presence of TB co-infection. Unlike HIV load, no such differences in mycobacterial load, as indicated by acid fast bacilli (AFB) staining, were observed in co-infected patients (data not shown).

While this reiterated similar observations by others in HIV-TB co-infected populations [12], to further evaluate the impact of viral loads on CD4+T cell levels in HIV-TB category, we divided HIV and HIV-TB populations into two groups, those with low viral load (≤10^5^ IU/ml) and high viral load (>10^5^ IU/ml). We then correlated the same with the CD4+T cell levels of the respective groups. Decrease in CD4+T cells directly correlates with HIV disease progression during HIV mono-infection [13]. We explored if such correlations were sustained during HIV-TB co-infection. As reported earlier, we also observed that the HIV patients with low viral load had high CD4+T cell counts and the patients with high viral load had low CD4+T cell counts (p<0.001). This correlation was absent in HIV-TB co-infected group (Fig. 1 C). HIV-TB patients with high viral load did not necessarily register depleted CD4+T cell counts. Additionally, high CD4+T cell counts in HIV-TB patients did not denote that the viral loads in these patients were low (Fig. 1C). One can postulate that during early stages of co-infection, macrophages activated by mycobacteria may contribute significantly to HIV turnover [14], [15], thereby increasing the total viral load without significant depletion of CD4+T cells. These observations clearly show that unlike patients with HIV infection, HIV disease monitoring on the basis of CD4+T cell counts in HIV-TB co-infected patients may be misleading.

### Assessment of Pro-inflammatory Environment in HIV-TB Patients Prior to Drug Therapies

Having seen that there is discordance in the CD4+T cell levels with viral loads in HIV-TB patients, the baseline immune status of all the study categories prior to the onset of treatments were documented. TNF-α is important in the containment of TB through granuloma formation and elimination of mycobacterial infection [16]. Low production and release of TNF-α in TB patients is hypothesized to be the reason behind the defective granuloma formation and hence impaired containment of mycobacterial infection [17]. We studied the plasma levels of TNF-α, IFN-γ, IL-12 p70 and IL-2 of HIV-TB category as an indicator of pro- or anti-mycobacterial environment in these patients. We further analyzed the data to check if the discordance exhibited between the CD4+T cell levels and viral loads in HIV-TB patients are also extended to their immune status.

**Figure 2:**
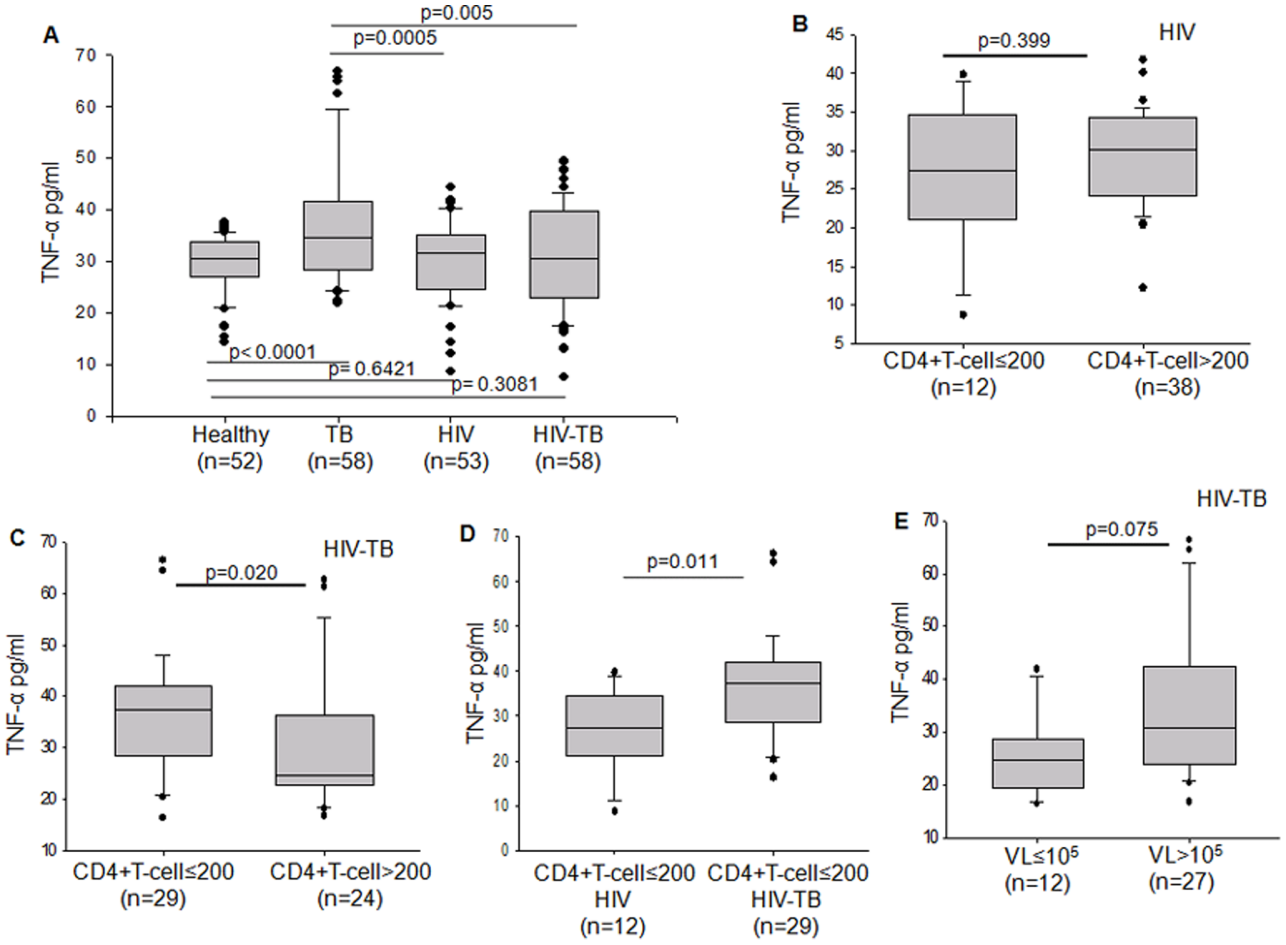
Plasma levels of TNF-α in relation to CD4+T cells and viral load. Box plots representing (A) comparison of plasma TNF-α levels between Healthy, TB, HIV and HIV-TB categories. (B) Plasma levels of TNF-α in HIV patients categorized on the basis of low CD4+T cells (≤200/mm^3^) and high CD4+T cells (>200/mm^3^). (C) Plasma levels of TNF-α in HIV-TB patients categorized on the basis of low CD4+T cells (≤200/mm^3^) and high CD4+T cells (>200/mm^3^). (D) Plasma levels of TNF-α in HIV and HIV-TB patients whose CD4+T cells are below ≤200/mm^3^ of blood. (E) Plasma levels of TNF-α in HIV-TB patients with low viral load (VL≤10^5^ IU/ml) and high viral load (VL>10^5^ IU/ml). The threshold for significance was set at p≤0.05. Bars above and below the plots represent the statistical significance (p value) between the groups.

Similar to other studies [18], TNF-α levels in the peripheral blood of the TB patients were higher than healthy category (p<0.0001), indicating a pro-inflammatory response to TB infection. However TNF-α levels were significantly less in both HIV patients (p = 0.0005) and HIV-TB co-infected patients (p = 0.005) when compared to TB patients (Fig. 2A).

CD4+T helper cells, primarily TH1 type, upon antigenic stimulation produces IFN-γ, which in turn potentiates the phagocytic activity of macrophages and the release of several pro-inflammatory cytokines including TNF-α [19]. In order to further understand that if the low TNF-α levels in HIV-TB category is not because of depleted CD4+T cells, the HIV and HIV-TB categories were further segregated into categories with low CD4+T cell levels (≤200 counts per mm^3^) and high CD4+T cell levels (>200 counts per mm^3^). Plasma TNF-α levels were not different in HIV patients with either high or low CD4+T cell levels (p = 0.399) (Fig. 2B). In HIV-TB category, patients with low CD4+T cell counts had higher levels of TNF-α (p = 0.020) (Fig. 2C). We then compared the TNF-α levels of HIV and HIV-TB patients having CD4+T cells ≤200/mm^3^. Despite low CD4+T cell levels, HIV-TB category maintained a significantly high level of TNF-α (p = 0.011) (Fig. 2D). With reports that macrophages in HIV patients are deficient in releasing adequate TNF-α upon *in-vitro* mycobacterial stimulation [20], one may speculate that other cells, apart from stimulated macrophages and lymphocytes, such as, dendritic cells, granulocytes, smooth muscle cells, eosinophils, mast cells, vascular endothelial cells etc. may contribute to the plasma TNF-α in HIV-TB patients and should be investigated further. We then verified if the viral loads in co-infected patients would have an impact on peripheral TNF-α levels. It was observed that HIV-TB population with higher viral loads had elevated levels of TNF-α, where upper quartile was 1.5 times higher in patients with viral load higher than 10^5^ IU/ml (Fig. 2E).

**Figure 3:**
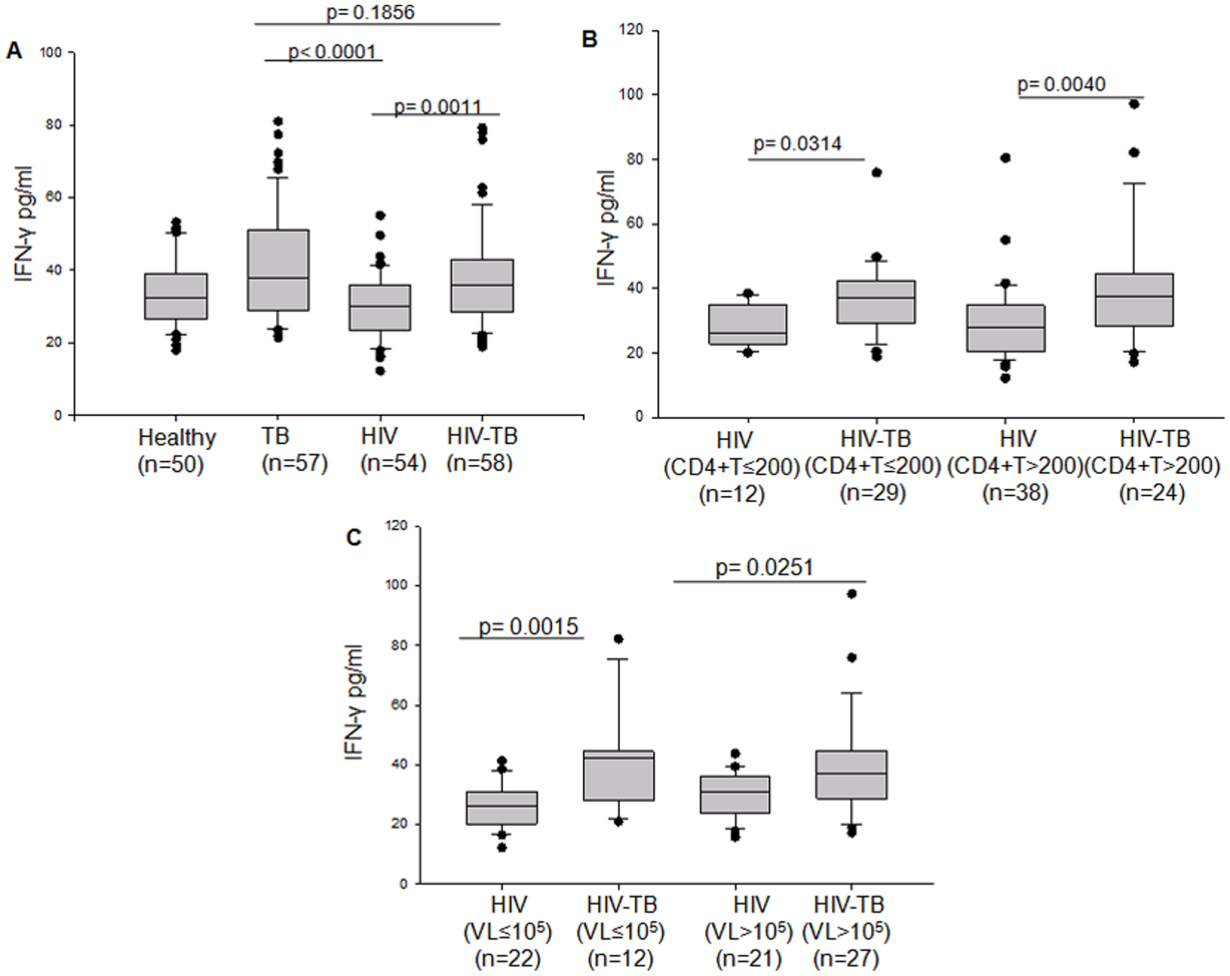
Plasma levels of IFN-γ in Healthy, TB, HIV and HIV-TB subjects and its relation to CD4+T cell and viral load under HIV and HIV-TB categories. Box plots representing (A) comparison of plasma IFN-γ levels among Healthy, TB, HIV and HIV-TB categories. (B) Plasma levels of IFN-γ in HIV and HIV-TB patients categorized on the basis of low CD4+T cells (≤200/mm^3^) and high CD4+T cells (>200/mm^3^). (C) Plasma levels of IFN-γ for HIV and HIV-TB patients with low viral load (VL≤10^5^ IU/ml) and high viral load (VL>10^5 ^IU/ml). The threshold for significance was set at p≤0.05. Bars above the plots represent the statistical significance (p value) between the groups.

CD4+T cells are considered to be the major source of IFN-γ. IFN-γ released from CD4+T cells plays a critical role in providing host the resistance to TB infection [21]. In our study, TB and HIV-TB categories showed higher levels of peripheral IFN-γ as compared to HIV category, possibly influenced by mycobacterial infection (Fig. 3A). Interestingly, the HIV-TB patients were able to maintain levels of IFN-γ almost equivalent to that of TB patients, despite a significant loss of CD4+T cells. Irrespective of low or high CD4+T cell counts or viral loads, HIV-TB patients always exhibited higher IFN-γ when compared to HIV patients (Fig. 3B and 3C). IFN-γ levels in HIV patients were lower than healthy controls, possibly because of the negative regulation by TH2 response [22] which is more common in HIV infection [6].

**Figure 4:**
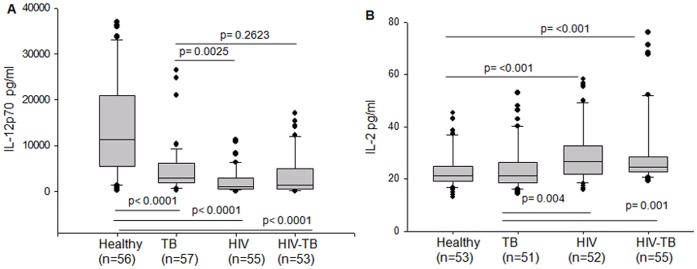
Blood plasma levels of cytokines Box plots representing blood plasma levels of (A) IL-12 p70, (B) IL-2 in the Healthy, TB, HIV and HIV-TB categories. The threshold for significance was set at p≤0.05. Bars above and below the plots represent the statistical significance (p value) between the groups.

IL-12 is a pro-inflammatory cytokine released from professional antigen presenting cells in response to antigenic stimulation. It is further involved in the differentiation of naive T cells into TH1 cells which in turn potentiate the release of interferon-gamma (IFN-γ) and hence is known to reduce the suppressive influence of anti-inflammatory cytokines, like IL-4 [23]. In our study, the healthy category maintained high levels of IL-12 p70 when compared with the categories having infections. Presence of high plasma levels of IL-12 p70 in healthy category was an unanticipated observation that we made in this population of randomly sampled healthy volunteers. Such high levels of IL-12 p70 were not seen in HIV, TB or HIV-TB categories. A fall in the levels of IL-12 p70 may directly correlate to the increased susceptibility towards an infection. HIV patients had very low levels of peripheral IL-12 p70 (Fig. 4A). Some studies have indicated the importance of IL-12 p70 in providing protection against HIV [24]. Moreover, IFN-γ and IL-12 p70 mutually influence their release [24], [25]. As IFN-γ levels were lower in HIV patients, this might be one of the explanations for decreased levels of IL-12 p70 in HIV when compared with TB and HIV-TB categories. However, this correlation between plasma levels of IFN-γ and IL-12 p70 were seen only in infected categories.

IL-2 cytokine provides a proliferative signal that is essential for growth and differentiation of the T cells into effector cells. We checked the levels of plasma IL-2 in our study population and observed that as compared with TB patients, both HIV and HIV-TB patients had higher levels of IL-2 (Fig. 4B). One might speculate that this can be a compensatory reaction of the depleted CD4+T cells of the host in HIV and HIV-TB patients.

On the whole, TB protective cytokines like TNF-α and IFN-γ were considerably higher in HIV-TB patients when compared with HIV infection alone. We presume that a mycobacterial background maintained high IFN-γ levels in HIV-TB category. IL-12 p70 levels in HIV-TB patients were, however, lower than that of TB patients. A recent study has indicated that lower levels of IL-12 in HIV patients precede susceptibility to tuberculosis in these patients [26]. An overall impression that emerges from the above results indicates that a pro-inflammatory immune environment is observed in HIV-TB category. At this juncture, it should be noted that though the pro-inflammatory environment in HIV-TB patient is seen; the depletion of effector cells (CD4+T cells) abates the immune system. Under these conditions, it is possible that these protective cytokines become potentially harmful leading to severe inflammatory responses and tissue damage in the host.

### Assessment of Anti-inflammatory Environment in HIV-TB Patients Prior to Drug Therapy

**Figure 5:**
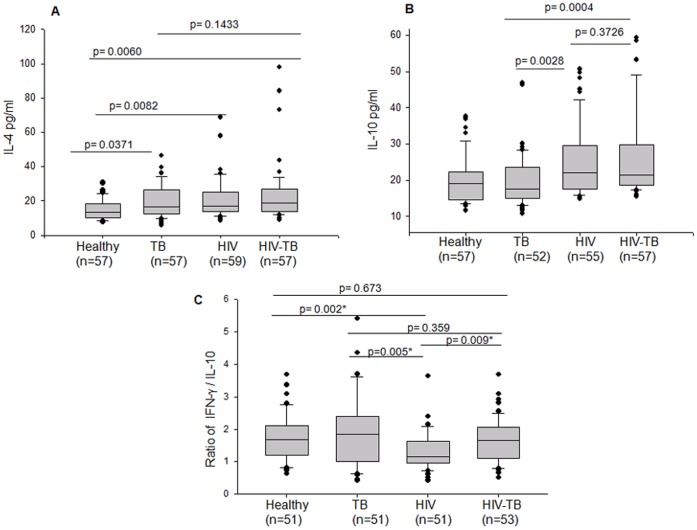
Comparison of anti-inflammatory cytokines Box plots representing plasma levels of (A) IL-4, (B) IL-10 (C) Ratio of IFN-γ/IL-10 in Healthy, TB, HIV and HIV-TB categories. The threshold for significance was set at p≤0.05. Bars above the plots represent the statistical significance (p value) between the groups.

To understand that the TH2 cytokines are important indicators of the magnitude and nature of the antigenic response to HIV in the presence or absence of mycobacterial co-infection; we scored for the peripheral levels of IL-4 and IL-10 in the newly diagnosed HIV-TB patients. The plasma IL-4 levels were significantly higher in all the patient categories as compared to the healthy, with the highest in HIV-TB category (Fig. 5A). The levels of IL-10 were significantly higher in HIV and HIV-TB categories as compared to Healthy and TB groups. Mycobacterial infection in HIV patients did not appear to influence the peripheral IL-10 levels in HIV-TB group (Fig. 5B).

### The Ratio of IFN-γ to IL-10 at the Time of Diagnosis of HIV-TB Co-infection

Once an assessment was made of the immune environment in all the study categories, we checked if the ratio of TH1 cytokine IFN-γ to the regulatory cytokine IL-10 (IFN-γ/IL-10) can be used as an indicator of the extent of TH1 or TH2 response during HIV-TB co-infection prior to any treatment. IFN-γ/IL-10 was a simplistic approach used to score for either TH1 or TH2 response in otherwise complicated immune scenario of HIV-TB co-infection. The ratios between pro- and anti-inflammatory cytokines have been used earlier in several studies [27], [28]. IFN-γ/IL-10 in different category of patients was compared (Fig. 5C). It should be made clear here that a ratio value near 1 does not mean that there is a balance between IFN-γ and IL-10. The baseline IL-10 plasma levels were, in general, lower than IFN-γ in all the categories, hence it was expected that the ratio will be always higher than 1. The median IFN-γ/IL-10 value for TB infected population was 1.836 (IQR: 1.039–2.379), for HIV infected population was 1.152 (IQR: 0.968–1.628), and for HIV-TB category, the values (median 1.646, IQR: 1.127–2.069) were intermediate between the values of TB and HIV categories. High values of IFN-γ/IL-10 in TB category indicated a profile dominated by TH1, low ratio suggested TH2 or regulatory environment in HIV patients, while an intermediate ratio clearly showed a compromised pro-inflammatory environment in HIV-TB patients. In a recent study, it has been indicated that IFN-γ/IL-10 is lower in HIV patient with multi-drug resistant TB as compared to TB patients [27]. Together with Skolimowska *et al* and our results, IFN-γ/IL-10 can, therefore, be further explored as a parameter to understand the immune status of HIV-TB co-infected patients.

### Co-occurrence of High Levels of IL-4 and TNF-α in HIV-TB Category Prior to Drug Therapy

While a pro-inflammatory immune response is both desired and observed during TB, HIV or HIV-TB infections, the influence of TH2 cytokines during co-infection is more controversial because they antagonize the effect of IFN-γ and IL-12 [29]. Increased IL-4 levels have been linked to the inhibition of bactericidal activities of macrophages and a poor outcome to anti-TB treatment [30]. It has been reported, with respect to tuberculosis that TNF-α can become potentially toxic to the host in the presence of IL-4 [31]. TNF-α toxicity in the presence of IL-4 may liquefy granuloma promoting relapse [32]. In our study, HIV-TB co-infected patients had higher levels of peripheral IL-4 (Fig. 5A) and also fairly high levels of peripheral TNF-α (Fig. 2A) when compared to HIV category. To further evaluate this, the cutoff values for TNF-α and IL-4 were selected on the basis of median values of the respective cytokines in healthy category. The levels of TNF-α higher than 29pg/ml and the levels of IL-4 higher than 13pg/ml were considered as co-occurrence of high TNF-α and IL-4. We observed that 34% of the HIV-TB population exhibited concurrent high levels of TNF-α and IL-4; of which 5 cases of TB relapse were registered after successful completion of TB treatment (Table S1). The IFN-γ/IL-10 ratio in the relapsed cases indicated an inclination for a pro-inflammatory environment (Table S1).

With high TNF-α and IFN-γ/IL-10 ratio existing simultaneously in HIV-TB patients before the onset of HAART, these patients are at a higher risk of TNF-α induced cytotoxicity and hyper-inflammation than patients with a single infection with HIV. According to the literature, the “paradoxical” symptomatic relapse of a prior infection despite a successful treatment is also an indication of IRIS [33]. Studies reveal that about 7–10% cases of patients on HAART develop TB-related IRIS in India [34]. In our study population, we had 9% cases amongst HIV-TB category showing this paradoxical symptom scored in the form of TB relapse. As TNF-α is known to cause tissue damage under certain conditions, we hypothesized that the HIV-TB patients exhibiting high TNF-α levels prior to any drug therapy would have a higher risk of TNF-α toxicity after initiation of HAART. Further, with IL-4 known to be involved in TNF-α toxicity and pulmonary fibrosis in TB patients [31], may also add to the TNF-α toxicity in HIV-TB category.

### Conclusion

Our study provides a comprehensive account of the baseline immune environment present in HIV-TB co-infected patients at the time of diagnosis that may have a bearing on HIV-TB pathogenesis. In our observations, there was an evident non-correlation between the CD4+T cell levels and viral loads in HIV-TB patients. An intermediate value of IFN-γ/IL-10 in HIV-TB group is suggestive of a compromised pro-inflammatory environment in HIV-TB patients as compared to TB patients. One of the significant observations of the study was the co-occurrence of high peripheral levels of a pro-inflammatory factor TNF-α and an anti-inflammatory factor IL-4 in 34% of HIV-TB patients at the time of diagnosis, 5 of which had a TB relapse after completion of TB therapy. In summary, the study culminates into two important observations (i) CD4+T-cell levels as baseline criteria to report the disease progression in terms of viral load in HIV-TB co-infected patients may be erroneous due to non-correlation of CD4+T cell levels and viral load in HIV-TB category and (ii) high plasma levels of TNF-α along with a high ratio of IFN-γ/IL-10 in HIV-TB category at the point of the detection of the diseases indicated a pre-dominant pro-inflammatory environment that may worsen upon HAART causing tissue damage and hyper-inflammation. The extension of these studies to larger and geographically distinct cohorts would help in deciding the baselines for these new cytokine markers which will have significant impact on the disease management of HIV-TB co-infected patients.

### Ethical Statement

All the study protocols were reviewed and approved by the independent institutional ethical committees of Mahavir Hospital and Research Center, Hyderabad; Blue Peter Public Health and Research Centre, Hyderabad and University of Hyderabad, Hyderabad, India. Informed written consents were taken from all the participants enrolled in the study.

## Materials and Methods

### Sample Collection

All the study protocols were reviewed and approved by the independent institutional ethical committees of Mahavir Hospital and Research Center, Hyderabad; Blue Peter Public Health and Research Centre, Hyderabad and University of Hyderabad, Hyderabad, India. Informed written consents were taken from all the participants enrolled in the study. The study population comprised of a total of 230 subjects recruited within the age group of 25–45 years during the period 2008 to 2011 (n = 230). The patients were recruited at Mahavir Hospital and Research Center, Hyderabad, India and Blue Peter Public Health and Research Centre, Hyderabad, India. 2–5 ml of blood was collected in EDTA treated vacutainers at the time of diagnosis of TB, HIV or HIV-TB before the start of any drug regimen. All patients selected for this study were newly diagnosed cases and had not been exposed to any drug therapy for either TB or HIV infection. These populations are, therefore, referred to as naïve, primary or newly diagnosed cases. The population was divided into four categories: TB patients (n = 57); seropositive HIV patients (n = 59); HIV-TB patients (n = 57) and asymptomatic healthy volunteers (n = 57). TB cases were confirmed by chest X-ray and sputum positive for Acid-fast bacilli (AFB) and extrapulmonary TB were diagnosed by granuloma biopsy. HIV infection was confirmed by TRIDOT/EIA COMB/COMBAIDS kit method. HIV-TB patients showed positive results for both HIV and TB tests. Asymptomatic healthy volunteers were found negative for TB and HIV tests and did not have any previous history of TB or any other major illness. Wherever required, the HIV and HIV-TB population was further divided on the basis of high and low CD4+T cell counts. The cut off value taken was 200/mm^3^ of blood as CD4+T cell ≤200/mm^3^ is considered as a parameter for the degree of immune compromisation [35]. Similarly, we have taken the median value of viral load 10^5^ IU/ml in HIV patients for defining population with high and low viral loads. Patients on treatment for any immuno-modulatory diseases by immunosuppressive or cortisols and pregnant women were excluded from the study group. TB, HIV and HIV-TB patients who have undergone respective drug treatment were also excluded. At the time of recruitment none of the subjects were under any medication.

### CD4+T Cell Count by FACS Analysis

CD4+T cells were counted by FACS using a monoclonal antibody cocktail of CD3-FITC/CD8-PE/CD45-PerCP/CD4-APC (MultiTEST, BD Biosciences, San Jose, CA). The samples were prepared according to the manufacturer’s protocol. 50 µl of whole blood was used for each analysis. The tubes were gently vortexed and incubated at room temperature in the dark for 15 minutes prior to acquiring the FACS data. Acquisitions and analyses were done using MultiSET software on FACS Calibur (BD Biosciences, San Jose, CA). The CD4+ T cell count was denoted in terms of counts per mm^3^ of blood.

### Viral Load Assay

Viral RNA was isolated using Nucleospin Viral RNA isolation kit (Machery-Nagel, Germany) from HIV and HIV-TB samples. The viral loads in terms of viral RNA copy numbers from plasma were estimated by using Artus HIV-1 RG RT-PCR kit (Qiagen, Germany) followed by real-time PCR RotorGene 3000 (Corbett Research Scientific, Australia) according to the manufacturer’s instruction. The minimum limit of viral RNA copy detection was 70 IU/ml. 1IU corresponds to 0.5 RNA copies/ml [calibrated using the international HIV standard (WHO)].

### Enzyme Linked Immunosorbent Assay (ELISA)

The cytokines were measured in blood plasma samples by sandwich ELISA using commercially available kits. ELISA assay for IL-12 p70, IL-2, IL-4, IL-10, TNF-α and IFN-γ were performed according to the manufacturer’s protocol (BD Biosciences San Jose, CA). The samples used for the ELISA were not freeze-thawed more than once. The absorbance was measured at 450 nm and 570 nm in ELISA reader (Biotek). The standard for different cytokines ranging from 2.5–5 to 100–500 pg/ml were plotted to generate a linear curve using four parameter regression formulas in GEN5 software. Sample concentration was calculated by standard curve.

### Statistical Analyses and Graphs

Statistical analyses were carried out using Sigma plot software version 11 and online tool from GraphPad (http://www.graphpad.com/quickcalcs/test1/). Mann-Whitney Rank Sum test was used to compare unpaired, parametric samples whereas the samples which passed the normality test i.e. the samples which exhibited normal distributions were compared using the unpaired Student’s t-test. The threshold for significance was set at p≤0.05. Results are represented as box plot where the upper quartile of the box represents the 75^th^ percentile and the lower quartile of the box represents the 25^th^ percentile. The line inside the box represents the median. The whiskers arising from either side of the upper half and the lower half of the box correspond to 1.5 times the interquartile range (IQR). Any datum to the further extreme of the whiskers is termed as outlier. Bars above the plots represent the statistical differences between the groups.

## Supporting Information

Table S1 **Co-occurrence of high TNF-α and IL-4 levels in HIV-TB co-infected patients.** Ten representative HIV-TB patients having high TNF-α and IL-4 with their CD4-T+ cell counts; viral loads and IFN-γ/IL-10. The TB relapse cases are marked.
(DOCX)

## References

[1] Whalen C, Horsburgh CR, Hom D, Lahart C, Simberkoff M, et al (1995) Accelerated course of human immunodeficiency virus infection after tuberculosis. Am J Respir Crit Care Med 151: 129–135. 7812542 10.1164/ajrccm.151.1.7812542

[2] Diedrich CR, Flynn JL (2011) HIV-1/mycobacterium tuberculosis coinfection immunology: how does HIV-1 exacerbate tuberculosis? Infect Immun 79: 1407–1417. 21245275 10.1128/IAI.01126-10 PMC3067569

[3] Sharma SK, Soneja M (2011) HIV & immune reconstitution inflammatory syndrome (IRIS). Indian J Med Res 134: 866–877. 22310819 10.4103/0971-5916.92632 PMC3284095

[4] Schluger NW, Rom WN (1998) The host immune response to tuberculosis. Am J Respir Crit Care Med 157: 679–691. 9517576 10.1164/ajrccm.157.3.9708002

[5] Weissman D, Poli G, Fauci AS (1994) Interleukin 10 blocks HIV replication in macrophages by inhibiting the autocrine loop of tumor necrosis factor alpha and interleukin 6 induction of virus. AIDS Res Hum Retroviruses 10: 1199–1206. 7848677 10.1089/aid.1994.10.1199

[6] Herbein G, Varin A (2010) The macrophage in HIV-1 infection: from activation to deactivation? Retrovirology 7: 33. 20380696 10.1186/1742-4690-7-33 PMC2859752

[7] Howard AD, Zwilling BS (1999) Reactivation of tuberculosis is associated with a shift from type 1 to type 2 cytokines. Clin Exp Immunol 115: 428–434. 10193414 10.1046/j.1365-2249.1999.00791.x PMC1905252

[8] Mellors JW, Munoz A, Giorgi JV, Margolick JB, Tassoni CJ, et al (1997) Plasma viral load and CD4+ lymphocytes as prognostic markers of HIV-1 infection. Ann Intern Med 126: 946–954. 9182471 10.7326/0003-4819-126-12-199706150-00003

[9] Subramanyam S, Hanna LE, Venkatesan P, Sankaran K, Narayanan PR, et al (2004) HIV alters plasma and M-tuberculosis-induced cytokine production in patients with tuberculosis. Journal of Interferon and Cytokine Research 24: 101–106. 14980074 10.1089/107999004322813345

[10] Mojtahedi Z (2012) Possible different serum IL-4 levels across populations: a reason for dissimilar incidence of Th1 and Th2 diseases. Med Hypotheses 78: 776–777. 22464042 10.1016/j.mehy.2012.03.003

[11] Al-Aska A, Al-Anazi AR, Al-Subaei SS, Al-Hedaithy MA, Barry MA, et al (2011) CD4+ T-lymphopenia in HIV negative tuberculous patients at King Khalid University Hospital in Riyadh, Saudi Arabia. Eur J Med Res 16: 285–288. 21810564 10.1186/2047-783X-16-6-285 PMC3353405

[12] Pawlowski A, Jansson M, Skold M, Rottenberg ME, Kallenius G (2012) Tuberculosis and HIV co-infection. PLoS Pathog 8: e1002464. 22363214 10.1371/journal.ppat.1002464 PMC3280977

[13] Hulgan T, Shepherd BE, Raffanti SP, Fusco JS, Beckerman R, et al (2007) Absolute count and percentage of CD4+ lymphocytes are independent predictors of disease progression in HIV-infected persons initiating highly active antiretroviral therapy. J Infect Dis 195: 425–431. 17205482 10.1086/510536

[14] Mariani F, Goletti D, Ciaramella A, Martino A, Colizzi V, et al (2001) Macrophage response to Mycobacterium tuberculosis during HIV infection: relationships between macrophage activation and apoptosis. Curr Mol Med 1: 209–216. 11899072 10.2174/1566524013363933

[15] Nakata K, Rom WN, Honda Y, Condos R, Kanegasaki S, et al (1997) Mycobacterium tuberculosis enhances human immunodeficiency virus-1 replication in the lung. Am J Respir Crit Care Med 155: 996–1003. 9117038 10.1164/ajrccm.155.3.9117038

[16] Lin PL, Plessner HL, Voitenok NN, Flynn JL (2007) Tumor necrosis factor and tuberculosis. J Investig Dermatol Symp Proc 12: 22–25. 10.1038/sj.jidsymp.5650027 17502865

[17] Takashima T, Ueta C, Tsuyuguchi I, Kishimoto S (1990) Production of tumor necrosis factor alpha by monocytes from patients with pulmonary tuberculosis. Infect Immun 58: 3286–3292. 2205576 10.1128/iai.58.10.3286-3292.1990 PMC313651

[18] Barnes PF, Fong SJ, Brennan PJ, Twomey PE, Mazumder A, et al (1990) Local production of tumor necrosis factor and IFN-gamma in tuberculous pleuritis. J Immunol 145: 149–154. 2113553

[19] Boehm U, Klamp T, Groot M, Howard JC (1997) Cellular responses to interferon-gamma. Annu Rev Immunol 15: 749–795. 9143706 10.1146/annurev.immunol.15.1.749

[20] Patel NR, Zhu J, Tachado SD, Zhang J, Wan Z, et al (2007) HIV impairs TNF-alpha mediated macrophage apoptotic response to Mycobacterium tuberculosis. J Immunol 179: 6973–6980. 17982088 10.4049/jimmunol.179.10.6973

[21] Green AM, Difazio R, Flynn JL (2013) IFN-gamma from CD4 T cells is essential for host survival and enhances CD8 T cell function during Mycobacterium tuberculosis infection. J Immunol 190: 270–277. 23233724 10.4049/jimmunol.1200061 PMC3683563

[22] Fenton MJ, Vermeulen MW, Kim S, Burdick M, Strieter RM, et al (1997) Induction of gamma interferon production in human alveolar macrophages by Mycobacterium tuberculosis. Infect Immun 65: 5149–5156. 9393809 10.1128/iai.65.12.5149-5156.1997 PMC175742

[23] Elser B, Lohoff M, Kock S, Giaisi M, Kirchhoff S, et al (2002) IFN-gamma represses IL-4 expression via IRF-1 and IRF-2. Immunity 17: 703–712. 12479817 10.1016/s1074-7613(02)00471-5

[24] Louis S, Dutertre CA, Vimeux L, Fery L, Henno L, et al (2010) IL-23 and IL-12p70 production by monocytes and dendritic cells in primary HIV-1 infection. J Leukoc Biol 87: 645–653. 20097848 10.1189/jlb.1009684

[25] Trinchieri G (1998) Interleukin-12: a cytokine at the interface of inflammation and immunity. Adv Immunol 70: 83–243. 9755338 10.1016/s0065-2776(08)60387-9

[26] Bordon J, Plankey MW, Young M, Greenblatt RM, Villacres MC, et al (2011) Lower levels of interleukin-12 precede the development of tuberculosis among HIV-infected women. Cytokine 56: 325–331. 21880503 10.1016/j.cyto.2011.08.018 PMC3466167

[27] Skolimowska KH, Rangaka MX, Meintjes G, Pepper DJ, Seldon R, et al (2012) Altered ratio of IFN-gamma/IL-10 in patients with drug resistant Mycobacterium tuberculosis and HIV- Tuberculosis Immune Reconstitution Inflammatory Syndrome. PLoS One 7: e46481. 23071578 10.1371/journal.pone.0046481 PMC3468619

[28] Sahiratmadja E, Alisjahbana B, de Boer T, Adnan I, Maya A, et al (2007) Dynamic changes in pro- and anti-inflammatory cytokine profiles and gamma interferon receptor signaling integrity correlate with tuberculosis disease activity and response to curative treatment. Infect Immun 75: 820–829. 17145950 10.1128/IAI.00602-06 PMC1828524

[29] van Den Broek M, Bachmann MF, Kohler G, Barner M, Escher R, et al (2000) IL-4 and IL-10 antagonize IL-12-mediated protection against acute vaccinia virus infection with a limited role of IFN-gamma and nitric oxide synthetase 2. J Immunol 164: 371–378. 10605032 10.4049/jimmunol.164.1.371

[30] Oswald IP, Gazzinelli RT, Sher A, James SL (1992) IL-10 synergizes with IL-4 and transforming growth factor-beta to inhibit macrophage cytotoxic activity. J Immunol 148: 3578–3582. 1588047

[31] Rook GA (2007) Th2 cytokines in susceptibility to tuberculosis. Curr Mol Med 7: 327–337. 17504117 10.2174/156652407780598557

[32] Fenhalls G, Wong A, Bezuidenhout J, van Helden P, Bardin P, et al (2000) In situ production of gamma interferon, interleukin-4, and tumor necrosis factor alpha mRNA in human lung tuberculous granulomas. Infect Immun 68: 2827–2836. 10768979 10.1128/iai.68.5.2827-2836.2000 PMC97494

[33] Shelburne SA, Visnegarwala F, Darcourt J, Graviss EA, Giordano TP, et al (2005) Incidence and risk factors for immune reconstitution inflammatory syndrome during highly active antiretroviral therapy. AIDS 19: 399–406. 15750393 10.1097/01.aids.0000161769.06158.8a

[34] Sharma SK, Dhooria S, Barwad P, Kadhiravan T, Ranjan S, et al (2010) A study of TB-associated immune reconstitution inflammatory syndrome using the consensus case-definition. Indian J Med Res 131: 804–808. 20571170

[35] Jung AC, Paauw DS (1998) Diagnosing HIV-related disease: using the CD4 count as a guide. J Gen Intern Med 13: 131–136. 9502375 10.1046/j.1525-1497.1998.00031.x PMC1496917

