# Supplementary material for: Discordance in CD4+T-Cell Levels and Viral Loads with Co-Occurrence of Elevated Peripheral TNF-α and IL-4 in Newly Diagnosed HIV-TB Co-Infected Cases
Source: PLoS One. 2013 Aug 1;8(8):e70250. doi: 10.1371/journal.pone.0070250 (PMC3731333; doi:10.1371/journal.pone.0070250)
Supplement: Table S1 — Co-occurrence of high TNF-α and IL-4 levels in HIV-TB co-infected patients. Ten representative HIV-TB patients having high TNF-α and IL-4 with their CD4-T+ cell counts; viral loads and IFN-γ/IL-10. The TB relapse cases are marked. (DOCX) [file pone.0070250.s001.docx]

**Table S1**:

| **Patient No.** | **TNF-α*** | | **IL-4*** | **CD4** | **Viral load** | **IFN-γ/IL-10** | **TB Relapse** |
| --- | --- | --- | --- | --- | --- | --- | --- |
| HIV-TB#1 | ++ | + | | 56 | 2914539 | 1.15 | No |
| HIV-TB#2 | ++ | + | | 85 | 607563 | 2.23 | **Yes** |
| HIV-TB#3 | + | ++++ | | 102 | 184108 | 1.64 | No |
| HIV-TB#4 | + | + | | 110 | 30449818 | 1.83 | **Yes** |
| HIV-TB#5 | ++ | +++ | | 110 | 1173392 | 1.36 | No |
| HIV-TB#6 | +++ | + | | 131 | 128513 | 2.80 | No |
| HIV-TB#7 | + | + | | 163 | 2142022 | 1.75 | **Yes** |
| HIV-TB#8 | ++ | +++ | | 248 | 253140 | 2.14 | No |
| HIV-TB#9 | + | ++ | | 605 | 5833 | 2.29 | **Yes** |
| HIV-TB#10 | + | + | | 627 | 2352920 | 1.16 | **Yes** |

* The cut off for TNF-α and IL-4 were selected on the basis of respective median values in Healthy controls.

~ 0.25-0.5 fold increase = +; ~ 0.5-1 fold increase = ++; ~ 1-1.5 fold increase = +++; above ~ 1.5 fold increase = ++++. IFN-γ/IL-10 ratios were above 1 in all the cases indicating inclination towards pro-inflammatory environment.
